# The Challenge of Bacterial Strain Identification: *Leptospira interrogans* Serovars Australis in a Dog and Long-Term Clinical Follow-Up

**DOI:** 10.3390/tropicalmed9120285

**Published:** 2024-11-22

**Authors:** Tommaso Furlanello, Elisa Mazzotta, Cristina Bertasio, Mario D’Incau, Laura Bellinati, Laura Lucchese, Alda Natale

**Affiliations:** 1 Veterinary Clinic and Laboratory San Marco, Via dell’Industria, 35030 Veggiano, Italy; tf@sanmarcovet.it; 2 Istituto Zooprofilattico Sperimentale delle Venezie (IZSVe), Viale dell’Università 10, 35020 Legnaro, Italy; lbellinati@izsvenezie.it (L.B.); llucchese@izsvenezie.it (L.L.); anatale@izsvenezie.it (A.N.); 3 National Reference Centre for Animal Leptospirosis (NRCL), Istituto Zooprofilattico Sperimentale della Lombardia e dell’Emilia Romagna “Bruno Ubertini”, Via Bianchi 7/9, 25121 Brescia, Italy; cristina.bertasio@izsler.it (C.B.); mario.dincau@izsler.it (M.D.)

**Keywords:** canine leptospirosis, chronic kidney disease, *Leptospira* Australis, sequencing, genotyping, serotyping

## Abstract

Leptospirosis is a widespread disease throughout the world, presenting in severe clinical forms in dogs. The pathogenicity of the different serovars in field infections is not fully documented, and clinical diagnosis is often limited to a combination of serological tests and molecular analyses. The latter, although a fundamental tool, cannot identify the infecting strain without further analysis. This study reports the use of various indirect (microscopic agglutination test, MAT) and direct (microbiological culture, real-time PCR) laboratory techniques, followed by typing protocols (Multi-locus Sequence Typing (MLST), Multiple Loci Variable number tandem repeat Analysis (MLVA), serotyping) that allowed for the identification of the *Leptospira* serovar Australis in a symptomatic and previously vaccinated dog (vaccine containing heterologous strains). This study reports long-term clinical follow-up (0–640 days) and describes the possible role of the infection in the development of chronic renal failure. This study aims to highlight how a combination of different techniques can be useful to better characterise the environmental circulation of zoonotic agents. Therefore, the identification and isolation of circulating *L.* strains would facilitate the updating of epidemiological data, enhance the knowledge of pathogenicity and long-term clinical effects, and provide a valuable resource for improving the efficacy of a specific serovar vaccination.

## 1. Introduction

Leptospirosis, a zoonotic disease caused by spirochetes belonging to the genus *Leptospira*, affects a wide range of mammals worldwide. The genus includes over 300 serovars based on the outer lipopolysaccharide antigens. However, serogroup classification can be confusing due to the overlapping antigenicity of different serovars [1]. Historically, diagnosis of leptospirosis has relied on the microscopic agglutination test (MAT), which uses reference strains to detect antibodies against infecting serogroups. The MAT has limitations in terms of specificity and reproducibility due to cross-reactivity among serovars and variability among diagnostic laboratories. The confirmation of infection requires a second serological sampling two-four weeks after the acute serum—not always available in clinical practice—which demonstrates seroconversion. In the convalescence period, cross-reacting IgM have been class switched and replaced by more serovar-specific IgG [2]. According to this immunological response developed in survivor animals, the characterisation of the infecting serogroup may be incorrect in the case of paradoxical reactions in acute-phase sera [3]. Molecular techniques are now widely adopted to detect *L.* spp. infection in blood and urine samples of clinical suspects, but they can usually recognise the presence of pathogenic strains without discrimination among species/serogroups. Furthermore, antibiotic treatments rapidly lead to a negative result in direct tools when the samples are collected after the beginning of the therapy [4].

Although leptospirosis is primarily characterised by acute tubulointerstitial nephritis and liver dysfunction, it can also affect multiple other organs, resulting in symptoms such as fever, pulmonary haemorrhage, uveitis, myositis, and reproductive failure [1]. While it is unclear whether specific serogroups are associated with different clinical presentations [1,3], the limitations of serological testing make it difficult to definitively link a particular serogroup to a specific set of symptoms.

As previously reported, in clinical settings, the diagnosis of leptospirosis is frequently a clinical diagnosis, supported by serological tests and/or molecular investigations. The identification of the strain of Leptospira that caused the disease in the subject is rarely conducted, except in experimental settings [1,5]. Leptospirosis is primarily investigated when acute symptoms are detected in clinical cases, while long-term consequences of the infection are rarely reported. The present case report describes a case of chronic renal failure as a consequence of severe renal disease and infection by *L. interrogans* sg Australis serovar Australis in a dog. This report underscores the significance of identifying the etiological agents of infectious diseases, both for the documentation of potential predispositions to chronic diseases and for the clarification of clinical developments. Furthermore, this case is noteworthy, as *L.* serogroup (sg) Australis is included in modern tetravalent vaccines and recently has been reported to be responsible for acute and severe disease in one dog [6]. It emphasises the necessity of implementing One Health surveillance and monitoring of zoonoses.

## 2. Materials and Methods

### 2.1. Anamnesis and Signalment

A 5-year-old male, intact Argentine Dogo was presented at the veterinary clinic (Veterinary Clinic and Laboratory San Marco, Veggiano (PD), Italy) with a history of severe vomiting and dysorexia, preceded by a few days of polyuria–polydipsia. The dog was usually confined to a fenced garden in a rural setting and had been vaccinated six months before the onset of the symptoms upon a veterinarian’s referral. The vaccine used was a commercial whole-cell bivalent vaccine for *Leptospira* Canicola and Icterohaemorrhagiae.

### 2.2. Laboratory Diagnostics

Serum, whole blood in K_3_EDTA, and urine were collected and analysed at the Istituto Zooprofilattico Sperimentale delle Venezie). Serum samples were tested by means of MAT, according to the WOAH method (Chap 3.1.12) [7]. The antigen panel included 8 serogroups and 9 serovars distributed by the Italian Reference Centre for Animal Leptospirosis as antigens in the routine diagnostic MAT (*L. interrogans* sg Australis serovar Bratislava; *L. interrogans* sg Canicola serovar Canicola; *L. kirschneri* sg Grippotyphosa serovar Grippotyphosa; *L. interrogans* sg Icterohaemorrhagiae serovar Copenhageni; *L. interrogans* sg Icterohaemorrhagiae serovar Icterohaemorrhagiae; *L. interrogans* sg Pomona serovar Pomona; *L. interrogans* sg Sejroe serovar Hardjo; *L. borgpetersenii* sg Tarassovi serovar Tarassovi; *L. borgpetersenii* sg Ballum serovar Ballum) [8]. The serum samples were tested at the screening dilution of 1:100. Samples with ≥50% agglutination were retested to determine an endpoint titre, using dilutions of serum beginning at 1:100 through to 1:12,800. Serum samples with the widely accepted minimum significant titre of 1:100 (reciprocal of the final dilution of serum with 50% agglutination) were assessed as positive.

*Leptospira* spp. DNA was investigated in blood and urine by means of a real-time PCR (rPCR) protocol, targeting a 87 bp genomic fragment within the rrs (16S) gene of pathogenic leptospires, as described in prior studies [9,10], using the TaqMan Universal PCR Master Mix (Thermo Fisher Scientific, Waltham, MA, USA). The samples were classified as positive if the threshold cycle (Ct) value was ≤38. *Leptospira* isolation was attempted from the urine sample via culture examination as previously described [7]. The isolated bacterial strain was genotyped at the National Reference Centre for Leptospirosis (Istituto Zooprofilattico Sperimentale della Lombardia e dell’Emilia Romagna) using Multi-locus Sequence Typing (MLST). The genotyping was performed using the 7-loci scheme proposed by Boonsilp in 2013 [11], which is based on the housekeeping genes glmU, pntA, sucA, tpiA, pfkB, mreA, and caiB, as previously described [9]. For allelic number and ST identification, the assembled and trimmed sequences were queried against the Bacterial Isolate Genome Sequence Database (BIGSdb), available on the *Leptospira* MLST website (https://pubmlst.org/leptospira/, accessed on 20th July 2015) sited at the University of Oxford [12]. The identification of ST permitted us to define the species of the *Leptospira* being tested and, through a comparison with the characterised isolates present in BIGSdb, to deduce the most probable serogroup and serovar. In addition, Variable Number Tandem Repeat (VNTR) analysis was performed on the bacterial isolate using the method proposed by Salaün et al. in 2006 on five discriminatory loci (VNTR-4, VNTR-7, VNTR-10, VNTR-Lb4 and VNTR-Lb5) [13,14]. The isolated strain was then characterised at serovar level using, at first, polyclonal sera for serogroup screening and, once the serogroup was identified, a monoclonal antibody panel specific for serogroup Australis—F81 C3, F81 C5, F81 C6, F81 C8, F90 C5, F90 C6, F90 C8, F90 C9. The monoclonal antibodies were acquired from WOAH Reference Laboratory for Leptospirosis—University Medical Centre, Amsterdam, The Netherlands [15,16,17].

## 3. Results

### Clinical Case Description

At the physical examination, the dog was hypothermic and depressed, with laboured breathing. A thoracic X-ray revealed diffuse pneumopathy with a moderate bronchial and interstitial pattern. An abdominal ultrasound reported that both kidneys had increased in size, with enhanced echogenicity of the renal cortical. The main abnormalities observed in the laboratory examination were systemic inflammation and severe renal failure. Clinical signs, creatinine levels, and systemic inflammation led to a diagnosis of acute kidney injury (AKI) and leptospirosis [18]. Thus, an appropriate antimicrobial treatment with intravenous ampicillin (Amplital-Vet^®^, Ceva Salute Animale S.p.A., Milano, Italia), along with supportive treatment as dictated by published guidelines, was established [18], pending the results of the serology and molecular analysis. Following a ten-day period of hospitalisation, the dog exhibited signs of partial improvement and was discharged with a prescription for continued medical treatment at home (Ronaxan^®^, Boehringer Ingelheim Animal Health Italia S.p.A., Milano Italia). In the meanwhile, serology reported positivity for *L.* interrogans sg Australis serovar Bratislava and *L.* kirschneri sg Grippotyphosa serovar Grippotyphosa (Table 1). *Leptospira* DNA was found in urine with a high Ct, while the blood tested negative. The dog was subjected to further monitoring of the liver and kidney status, with progressive improvement of renal function.

The isolation from urine was successful; serotyping tests on bacterial isolates identified the strain as serogroup Australis, serovar Australis. Genotyping analysis confirmed that the strain belonged to ST 198 (*L.* interrogans Australis Australis) with a VNTR pattern of 2-10-10-neg-5 for the loci 4bis-7bis-10bis-Lb4-Lb5, respectively.

These results are summarised in Table 1.

Following a period of almost two years since the initial diagnosis of AKI due to leptospirosis, the dog was presented for further consultation. The symptoms exhibited were similar to those observed at the time of the first event but had become chronic and unresponsive to a repetition of the treatment with oral doxycycline, as prescribed by the referral veterinarian. The dog reported a 20% loss in body weight, along with an indication of severe depression and dehydration and a low rectal temperature (37.3 °C). Haematology and biochemical analyses highlighted severe renal failure, and an abdominal ultrasonography reported decreased kidney length and increased cortical thickness. Serology had been repeated, reporting positive titres for *L.* Australis serovar Bratislava (1:1600), *L.* Canicola serovar Canicola (1:100), *L.* Grippotyphosa serovar Grippotyphosa (1:400), *L.* Icterohaemorrhagiae serovar Copenagheni (1:200), and *L*. Pomona serovar Pomona (1:400). Due to the severity of the clinical condition, the lack of improvement following a second medical treatment, and the chronic nature of the illness, the owner refused further analysis and opted for euthanasia a few days after the evaluation. Unfortunately, an autopsy was not allowed. The most relevant laboratory findings are reported in Table 2.

## 4. Discussion

This clinical case offers several points of interest. The pattern of seroreactivity identified by MAT serology can predict the infecting serogroup but not the serovar nor the genospecies. Different genotypes can have a similar serological pattern (and vice versa); thus, accurate identification of the sources of infection requires sequence typing [1]. In this case report, different laboratory techniques allowed for a precise diagnosis of the etiological agent and made it possible to describe a case of *Leptospira* sg Australis serovar Australis natural infection. Previous experimental studies on the pathogenicity of serovar Bratislava, also belonging to serogroup Australis, presented conflicting results. In the first experimental study, no leptospiraemia, urinary shedding, or clinical disease were detected following the infection [19]. However, subsequent studies successfully induced the clinical disease using a serovar Bratislava strain [20].

Leptospirosis in dogs can manifest with signs of vasculitis, acute kidney injury, and/or hepatic injury, which are variable based on infecting strain and host immune response. Other clinical presentations can include fever, pulmonary haemorrhage, uveitis, myositis, and reproductive failure [20]. In our case, we mainly observed that an AKI and pulmonary signs, with a haemorrhagic syndrome, have appeared as a more and more frequent, life-threatening complication of canine leptospirosis in some areas of Europe [21]. Pulmonary complications could be a peculiar characteristic of *L.* Australis infections, since it was described in experimental cases as well [22]. *Leptospira* infections are a common cause of acute renal disease, and, during the acute phase of infection, the predominant renal lesions are those of acute interstitial nephritis, with tubular cell necrosis, apoptosis, and regeneration [23]. As previously reported, the most common clinicopathologic findings in dogs with *L.* Australis infection identified multiple organ damage with consistent renal involvement, as well as muscle, heart, and liver impact [24]. In the majority of symptomatic dogs, clinicopathologic evidence of nephropathy mirrored histologically apparent interstitial damage and was characterised by low urine-specific gravity, glycosuria, proteinuria, and cast formation. Similar findings were reported in previous studies in which infections were caused by serogroups other than Australis [1,25].

In this present case, the organ most affected was the kidney, with a severe form of renal failure, associated with severe systemic inflammation, as witnessed by the increase in C reactive protein, the main acute phase protein in dogs. According to previous studies in the literature, the dog reported mild alteration of liver enzyme activities, dominated by increased ALT and total bilirubin, reflecting mild liver involvement and damage [1]. Increased blood urea nitrogen, creatinine, and phosphate concentrations are associated with acute kidney injury [18]. At the time of the first presentation, the antibacterial treatment, along with appropriate management of the renal injury, particularly with intensive fluid therapy, was effective for reducing the clinical symptoms. After two months, the inflammation ceased, but the dog maintained mild hyperazotemia, suggesting that the recovery, as expected, was incomplete. Precautionary oral treatment with doxycycline did not prevent the recurrence of kidney failure and the final deterioration of the clinical condition.

The long follow-up in our case allowed us to describe chronic kidney disease (CKD) linked to *L.* Australis infection in an Argentine Dogo. Possible pathogenic mechanisms are (i) chronic tubulointerstitial inflammation caused by acute leptospirosis following recovery from AKI, (ii) an incomplete clearance of the renal tubules, and (iii) re-infection due to exposure to an environment contaminated with *Leptospira* strains. This mechanism may be related to the carrier status of leptospires in the kidneys, because a few studies have reported that the bacteria remain latent in the kidney for a long time [26]. As a limitation, in this case, we were unable to perform urine PCR to assess the possible persistence of leptospiruria despite treatment with doxycycline [1,20,27]. Nevertheless, the direct research analysis of Leptospira in this case may have yielded inaccurate information because, as previously reported, following the onset of an acquired immune response in the host, leptospires are eliminated from the bloodstream, although they may persist in the form of a biofilm within the eye or renal tubules. The progression of tubulointerstitial nephritis to renal fibrosis has been documented in dogs [28,29,30]. Therefore, we cannot establish whether the kidney’s degeneration was caused by persistent bacterial proliferation or was secondary to a maladaptive repair process, which resulted in progression to CKD. Maladaptive renal repair after AKI has been implicated in CKD progression because it can lead to inflammation, which promotes immune cell infiltration, followed by interstitial fibrosis [26]. The renal tubules and tubulointerstitium are the main components of the kidney that respond to injuries; hence, tubular epithelial cells play important roles in driving the progression of AKI to CKD. The precise pathogenic mechanism of leptospirosis is not fully understood. It targets the kidneys, evading the immune system. The disease is characterised by kidney damage, caused by the binding of LipL32 to toll-like receptor 2, which triggers an inflammatory cascade [26]. The seropositivity at the time of the follow-up might have been compatible with a chronic infection, a condition that had been described in some dogs, after appropriate antimicrobial treatment [27], connected to persistently high serological antibodies against *Leptospira* [1,31], or due to reinfection after environmental exposure [32,33]. The duration and potential spread of shedding is uncertain and may depend on the serovar. Indirect transmission occurs when animals or humans are exposed to a contaminated environment where organisms persist in the urine of an infected host. Water is the main means of spread, with slow-moving warm water favouring survival [34]. Further studies are needed to understand whether serogroup Australis, or some serovars in this group, are more capable of causing severe chronic kidney disease than other serogroups. Isolation of the bacteria from clinical specimens makes it possible to serotype and fully characterise the infecting strain, improving our understanding of the epidemiology of leptospirosis [35], although, nowadays, MLST permits the definition of ST, even on DNA extracted from biological samples.

It is widely acknowledged that the correlation between the infecting leptospiral strain and the clinical manifestations of disease is independent of the involved serovar [1]. Nevertheless, the routine utilisation of a real-time PCR with primers that are common to the 17 pathogenic *Leptospira* species and encompass over 300 different serovars does not permit the identification of the serovar responsible for the clinical symptoms of subjects with leptospirosis. A novel approach is currently being proposed [9,36], with the potential to resolve a debate that has been ongoing for over four decades. The debate concerns the possibility that distinct clinical syndromes are associated with specific serogroups and serovars [3]. The utilisation of different laboratory techniques to characterise the infecting strain in the dog permitted its definition with complete certainty. Upon serological diagnosis by MAT on the blood sample, the reaction toward the Bratislava serovar demonstrated that it belonged to the serogroup Australis. The MLST analysis of DNA extracted from the bacterial culture reported results indicative of *L.* serovar Australis ST 198. The identification was confirmed by classical serotyping, performed with monoclonal antibodies on the isolated strain. AKI associated with *L.* var Australis has been reported as the most common feature in symptomatic dogs [37]; in our case, it was shown to lead to the development of chronic renal damage. This finding highlights the importance of long-term monitoring of veterinary patients who have contracted leptospirosis and present with a severe clinical form. Given the complexity and severity of the symptomatic pictures in animals vaccinated with products that reduce the impact of the clinical form only for certain serovars (no cross-protection among serogroups), it is imperative to assess the effective protection offered by the vaccines commercially available.

## 5. Conclusions

This study reports severe clinical symptoms associated with *L.* Australis infection in a dog vaccinated with a commercial bivalent vaccine against leptospirosis and highlights the importance and complementarity of the different diagnostic techniques.

Furthermore, this study posits that severe clinical forms of Australis serogroup infection in dogs may result in long-term consequences (chronic kidney failure). In the recent past, the attribution of clinical syndromes to a serovar rather than to another was based on the serological response to the representative serovar included in the diagnostic MAT panel (e.g., Bratislava instead of Australis). A comprehensive characterisation of the strain, employing either MLST or isolation in culture followed by serotyping, is essential for the collection of punctual data and the accurate association of specific syndromes with corresponding *L.* strains. As a result, the selection of strains for vaccine development can be evaluated in an optimal manner.

This study emphasises the importance of isolation methods despite increased time commitment. Isolation, when successful, allows for the identification and characterisation of the strain by both molecular and serological methods, even in samples that yield bacterial DNA that is too low for direct molecular characterisation. The identification of field strains and their isolation would streamline the updating of epidemiological data about circulating strains, facilitate the correlation with possible long-term pathologies and their prognosis, and highlight the challenging necessity of implementing vaccination protection for specific serovars.

## Figures and Tables

**Table 1:** MAT titres, molecular findings, and typing (MLST and VNTR) findings at the acute onset of the clinical signs of leptospirosis in the dog.

Analysis	Biological Sample	Result							
MAT	Serum	*L. interrogans* sg Australis serovar Bratislava				1:800			
		*L. kirschneri* sg Grippotyphosa serovar Grippotyphosa				1:200			
*Leptospira* spp. rPCR	Urine	Positive (Ct 36.6)							
	Blood	Negative							
*Leptospira* spp. isolation	Urine	Positive							
Typing MLST	Bacterial strain	glmU	pntA	sucA	tpiA	pfkB	mreA	caiB	ST
		1	66	2	1	5	3	4	198
Typing VNTR	Bacterial strain	4bis		7bis	10bis		Lb4		Lb5
		2		10	10		Neg		5
Serotyping	Bacterial strain	*L. interrogans* sg Australis serovar Australis							

Neg: negative. MAT: microagglutination test; rPCR: real-time PCR; MLST: Multi-locus Sequence Typing; VNTR: Variable Number Tandem Repeat.

**Table 2:** Selection of the most relevant laboratory data, from the day of accession to the last evaluation available, realised a few days before the euthanasia.

Laboratory Test [Reference Intervals]	0	+2	+5	+10	+14	+30	+60	+640
Serum paraoxonase (PON-1) [3.86–5.53 IU/L]	2.57	1.93	2.94	2.25	1.86	3.28	3.12	2.77
C Reactive Protein [0.01–0.22 mg/dL]	4.13	3.63	1.64	1.15	1.75	0.34	0.44	2.57
ALT [22–78 IU/L]	58	69	84	91	101	38	33	38
Total Bilirubin [0.11–0.31 mg/dL]	0.37	0.36	0.51	0.42	0.31	0.24	0.20	0.29
Urea [16–49 mg/dL]	374	419	297	168	51	51	38	315
Creatinine [0.83–1.42 mg/dL]	8.47	8.62	4.55	3.54	2.57	2.41	2.05	14.65
Phosphorus [2.3–5.0 mg/dL]	13.3	14.6	10.8	7.0	6.1	6.5	6.1	17.5

ALT: alanine aminotransferase.

## Data Availability

The datasets generated during and/or analysed during this current study are available from the corresponding author on reasonable request.
